# What constitutes high risk for venous thromboembolism? Determining an appropriate threshold to initiate prophylaxis

**DOI:** 10.1016/j.jtha.2026.02.004

**Published:** 2026-03-04

**Authors:** Benjamin G. Mittman, Bo Hu, Phuc Le, Matthew A. Pappas, Aaron Hamilton, Michael B. Rothberg

**Affiliations:** 1 Medical Scientist Training Program, School of Medicine, Case Western Reserve University, Cleveland, Ohio, USA; 2 Center for Value-Based Care Research, Department of Internal Medicine and Geriatrics, Primary Care Institute, Cleveland Clinic, Cleveland, Ohio, USA; 3 Department of Population and Quantitative Health Sciences, School of Medicine, Case Western Reserve University, Cleveland, Ohio, USA; 4 Department of Biostatistics and Bioinformatics, Duke University, Durham, North Carolina, USA; 5 Department of Hospital Medicine, Integrated Hospital Care Institute, Cleveland Clinic, Cleveland, Ohio, USA

**Keywords:** clinical decision support, decision analysis, hemorrhage, mathematical model, venous thromboembolism

## Abstract

**Background:**

Guidelines recommend pharmacological venous thromboembolism (VTE) prophylaxis only for high-risk patients, but the probability of VTE that is considered “high risk” is not specified.

**Objectives:**

We compared cost-effectiveness analysis (CEA) with alternative approaches to define an appropriate probability threshold for initiating prophylaxis in medical inpatients.

**Methods:**

Our analytic sample included 47 889 adults admitted to 10 Cleveland Clinic hospitals from October 2017 to January 2020, as well as 214 hospitalists and internal medicine residents who were surveyed from June to November 2023. We compared decision analysis with 4 alternative approaches to determine a probability threshold: the Youden Index, deriving a probability from a widely used point-based model, asking physicians’ opinions, and observing physician behavior. To assess each unique approach’s threshold, we applied the Cleveland Clinic VTE Model to calculate the percentage of patients whose predicted risk exceeded that threshold, and we modeled hypothetical adverse events (VTE plus major bleeding) if that threshold were used to guide prescribing.

**Results:**

Most thresholds ranged from 0.9% to 1.5%, inversely corresponding to 19% to 35% of patients being high risk. The CEA threshold of 1% would result in 15.9 adverse events per 1000 individuals. The lowest threshold, 0.3%, produced the fewest adverse events but was highly inefficient. Compared with CEA, the 0.3% threshold required treating more than 1000 patients to prevent 1 adverse event, and quadrupled the prophylaxis rate (100% vs 24.9%).

**Conclusion:**

To improve the efficiency of prophylaxis and standardize care, guidelines should explicitly quantify the high risk of VTE using a standardized probability threshold range.

## 1 | INTRODUCTION

Venous thromboembolism (VTE) affects 300 000 to 600 000 people and causes up to 100 000 deaths each year in the United States, with at least half of all cases attributable to current or recent hospitalization [1–4]. Randomized controlled trials in medical inpatients have demonstrated that prophylaxis with low-molecular-weight heparin (LMWH) can reduce symptomatic VTE [5–8]. However, LMWH increases rates of heparin-induced thrombocytopenia and bleeding [9,10], rendering indiscriminate use harmful and expensive. Therefore, major guidelines all recommend pharmacological prophylaxis for medical inpatients only if they are at high risk of VTE [11–14].

However, none of these guidelines defines the *probability* of VTE that should be considered high risk. Instead, high-risk patients are described as a category based on risk factors or care settings. Some guidelines recommend scoring systems, but these point-based systems do not quantify the probability of VTE [15,16]. This lack of clarity may promote clinician-level variation in VTE prophylaxis [17] and contribute to overuse [18–20]. Establishing an accepted probability threshold for prophylaxis could help reduce variation and improve the quality of care.

Decision analysis is the most comprehensive approach for determining an optimal probability threshold [21,22]. A decision-analytic model compares the expected value of a decision based on the outcomes, which are assigned utilities and costs, weighted by their probability of occurrence [21]. A major limitation of these models is that they incorporate a complex set of assumptions and modeling choices. If clinicians do not know the structure of a model or do not trust it, they are less likely to follow its recommendations [23]. For these reasons, models are often more useful for gaining insight into a problem than for determining exact thresholds.

There are other approaches to selecting a threshold. These often produce arbitrary thresholds without considering potential implications. For example, the Padua model’s threshold of ≥4 points was selected to maximize discrimination between low- and high-risk patients [16], but discrimination is not necessarily the most important factor in a decision. Worse yet, its discrimination was not reproducible in other cohorts [24,25]. The best approach to selecting a threshold is to balance the benefits and harms of prophylaxis and align with stakeholders’ (patients and clinicians) priorities.

We assessed 4 empirical and theoretical approaches for determining a probability threshold for VTE prophylaxis and compared each with a published decision-analytic cost-effectiveness model. For each approach, we computed the probability threshold and the corresponding percentage of patients who would be considered high risk and thus potentially eligible for pharmacological prophylaxis. We created a model of prophylaxis-guided decision support to estimate rates of adverse events—VTE and major bleeding (MB)—expected for the threshold derived from each approach. We then compared the efficiency of different threshold-based approaches by measuring the relationship between prophylaxis rates and the expected adverse event rates at each threshold.

## 2 | METHODS

### 2.1 | Setting and participants

We studied 4 approaches to determining a probability threshold and the implications of each threshold. Each approach uses a different conceptual or statistical method to derive a probability threshold that can subsequently be used for risk stratification. Certain thresholds were derived from a statistical model that can also serve as a risk assessment model, but this was not a requirement for any approach. We compared each threshold with a comparator threshold based on risk stratification results and expected clinical outcomes.

We included physician and patient samples from 10 Cleveland Clinic hospitals in Ohio and Florida. Hospitals varied in size from a 126-bed community hospital to a 1400-bed quaternary care academic medical center. The physician sample included internal medicine residents and hospitalists surveyed from June to November 2023. The patient sample consisted of medical patients aged ≥18 years admitted from October 2017 to January 2020. Patient data were extracted from the electronic health record system and verified for accuracy and completeness. Statistical analyses were performed using R version 4.2.3 (R Foundation for Statistical Computing). All data collection and analysis procedures adhered to the Strengthening the Reporting of Observational Studies in Epidemiology guidelines [26] for observational studies (Supplementary Table S1). The study was approved by the Cleveland Clinic Institutional Review Board (numbers 22–321 and 14–240).

### 2.2 | Variables

Analytic variables included 14-day hospital-acquired VTE, in-hospital MB, prophylaxis use, the Padua score [16], the probability of VTE determined by the Cleveland Clinic VTE Model (CCVM) [27], and the probability of MB determined by the Cleveland Clinic Bleeding Model (CCBM) [28]. VTE, MB, and prophylaxis were binary outcomes. Padua scores ranged from 0 to 20 points [16], and both the CCVM and CCBM probabilities ranged from 0 to 1.0. VTE and MB outcomes were previously identified and validated during the development and validation of the CCVM [27] and CCBM [28]. Prophylaxis was identified from the medication administration record and included subcutaneous heparin 5000 Units 2 to 3 times daily, enoxaparin 40 mg daily, dalteparin 5000 Units daily, and fondaparinux 2.5 mg daily.

Variables used to compute patients’ Padua scores and CCVM and CCBM probabilities were assessed for missingness, and any variable with ≥5% missing data was assumed to be missing at random. For variables with <5% missing data, we excluded patients who were missing any of those variables. Multiple imputation with the random forest method (implemented in the *missForest* R package [29]) was used to impute values assumed missing at random, based on the most clinically relevant and strongly correlated variables identified in univariate analyses (see Supplementary Methods).

### 2.3 | Comparator: decision analysis

Decision-analytic models compare the expected value of a decision based on its expected outcomes. We used a published model of VTE prophylaxis [30]. Each outcome was assigned a cost and a utility, and weighted by its probability of occurrence. Prophylaxis costs included 5 days of enoxaparin, plus nursing and pharmacy costs. Costs of complications (eg, MB) were based on additional hospital days incurred. Posthospitalization VTE costs included readmission, 3 months of warfarin, and international normalized ratio tests. A small disutility was applied to prophylaxis for potential complications and the pain of injection. Other utilities were based on length of stay and complications of major or minor bleeding and heparin-induced thrombocytopenia. Full details of the model structure and parameters appear in the original publication [30].

We selected 2 thresholds generated by the model. The first was the value at which the cost-effectiveness of prophylaxis was exactly $100 000/quality-adjusted life year (QALY), because probabilities of VTE above that threshold would be “cost-effective” based on a generally accepted willingness to pay. Thus, high-risk patients would be those for whom prophylaxis was cost-effective. The second threshold represented the point at which the expected value of prophylaxis in QALYs exactly equaled the expected value of no prophylaxis. In this scenario, high-risk patients were those expected to derive any net benefit from prophylaxis, regardless of cost.

### 2.4 | Approach 1: maximize sensitivity and specificity of a logistic regression model (Youden Index)

The CCVM is a validated prediction model that computes personalized VTE risk in medical patients based on the most important risk factors [27]. The model was developed from approximately 155 000 Cleveland Clinic patients and has been externally validated [27]. Using the most recent version of the model (see Supplementary Table S2 for details), we calculated VTE probabilities for all patients in the current study. The Youden Index [31], derived from the receiver operating characteristic curve, summarizes the overall accuracy of a binary prediction model and identifies a threshold value that maximizes the sum of sensitivity and specificity. We selected the CCVM’s Youden Index as the probability threshold.

### 2.5 | Approach 2: derive a probability from a point-based model (Padua)

The Padua score is a validated risk assessment model derived from medical inpatients in Padua, Italy [16]. The score is calculated by assigning points to risk factors and summing them. A score of 4 or more is considered high risk. To convert this score to a probability, we measured the observed VTE rate among patients in our cohort who had a score of 4.

### 2.6 | Approach 3: survey physicians

We elicited physicians’ stated thresholds directly via a survey. We asked 2 questions: (1) “What probability of developing VTE during hospitalization would you consider high risk?” and (2) “What is the largest number of patients that you would be willing to give prophylaxis to in order to prevent one VTE?” Question 1 (Q1) included a range from 0% to 10%, and question 2 (Q2) was a free-text response. Q1 directly assessed physicians’ threshold, whereas Q2 assessed it indirectly.

In Q2, we excluded blank responses, text answers that did not correspond to a number, and values <1, which suggested the question was misunderstood. We also excluded 2 overly influential values (2000 and 1 000 000) identified as outliers. For each question, we computed the mean, median, and range of eligible values. To calculate a threshold for Q2, we divided 1 by the number needed to treat (NNT), then divided the result by LMWH’s estimated efficacy (46%) in preventing VTE [7,32].

### 2.7 | Approach 4: examine physician behavior

To determine the implied threshold based on physician behavior, we identified the predicted probability of VTE at which 50% of patients in our sample received prophylaxis, suggesting equipoise among physicians. We did this by fitting a simple logistic regression model, with the CCVM-predicted risk as the independent variable and prophylaxis receipt as the outcome. We then used the model coefficients to calculate the predicted risk corresponding to a 50% probability of receiving prophylaxis.

### 2.8 | Applying the risk thresholds

For each approach, we calculated the percentage of patients whose predicted VTE probability, based on the CCVM, would be at or above the threshold and therefore “high risk.” We plotted thresholds against the percent of high-risk patients and compared each with the cost-effectiveness analysis (CEA) threshold. Next, we modeled the number of adverse events (VTE and MB) that would be expected if patients were treated under each of these thresholds. Adverse events were predicted based on published relative risk estimates from meta-analyses of randomized controlled trials [12,33]. We first calculated the expected number of adverse events in our cohort, assuming no prophylaxis (the base rates), using the cohort’s mean predicted CCVM and CCBM risks. We then used published estimates of the relative risk of VTE (0.54) [12,33] and MB (1.65) [33] with heparin compared with placebo to calculate the expected number of events at each threshold, given the corresponding prophylaxis rate.

In addition, because guidelines recommend withholding prophylaxis from patients at high risk of MB, and our goal was to simulate guideline-concordant prophylaxis, we assumed that patients at high risk of MB according to the CCBM [28] would not receive prophylaxis. Therefore, we estimated prophylaxis at 100% for patients with a high VTE risk and low MB risk, and at 0% for all other patients. Expected VTE and MB events were calculated according to the formulas:

$$E_{VTE}=\left(AV_{\left[HV,LB\right]}\times N_{\left[HV,LB\right]}\times RR_{VTE}\right)+\left(AV_{\left[LV,LB\right]}\times N_{\left[LV,LB\right]}\right)+\left(AV_{\left[HB\right]}\times N_{\left[HB\right]}\right)$$

$$E_{MB}=\left(AB_{\left[HV,LB\right]}\times N_{\left[HV,LB\right]}\times RR_{MB}\right)+\left(AB_{\left[LV,LB\right]}\times N_{\left[LV,LB\right]}\right)+\left(AB_{\left[HB\right]}\times N_{\left[HB\right]}\right)$$

where *E* = expected number of VTE or MB events; *RR* = relative risk of VTE or MB with heparin vs placebo; *AV* = average VTE risk; *AB* = average bleeding risk; *N* = number of patients; [*HV, LB*] denotes patients with a high VTE risk and a low bleeding risk; [*LV, LB*] denotes patients with a low VTE risk and a low bleeding risk; and [*HB*] denotes patients with a high bleeding risk and any VTE risk. We expressed events as an expected rate per 1000 individuals (see Supplementary Table S3 for modeling calculations).

We compared expected combined adverse event (VTE plus MB) rates across probability thresholds. To achieve this, we first plotted the prophylaxis rate for eligible patients against the corresponding adverse event rate at each threshold. Then, for each threshold, we calculated the additional number of patients who would need prophylaxis to prevent 1 additional adverse event relative to the next-highest threshold. We defined this value as the incremental NNT, which was calculated according to the formula:

$$NNT=\frac{\left(P_{T2}-P_{T1}\right)}{\left(E_{T1}-E_{T2}\right)}$$

where *P* = the threshold-guided rate of prophylaxis per 100 eligible patients; *E* = the expected rate of adverse events per 100 individuals; [*T*_1_] denotes the comparator threshold; and [*T*_2_] denotes the baseline threshold.

## 3 | RESULTS

### 3.1 | Physician and patient samples

**FIGURE 1:**
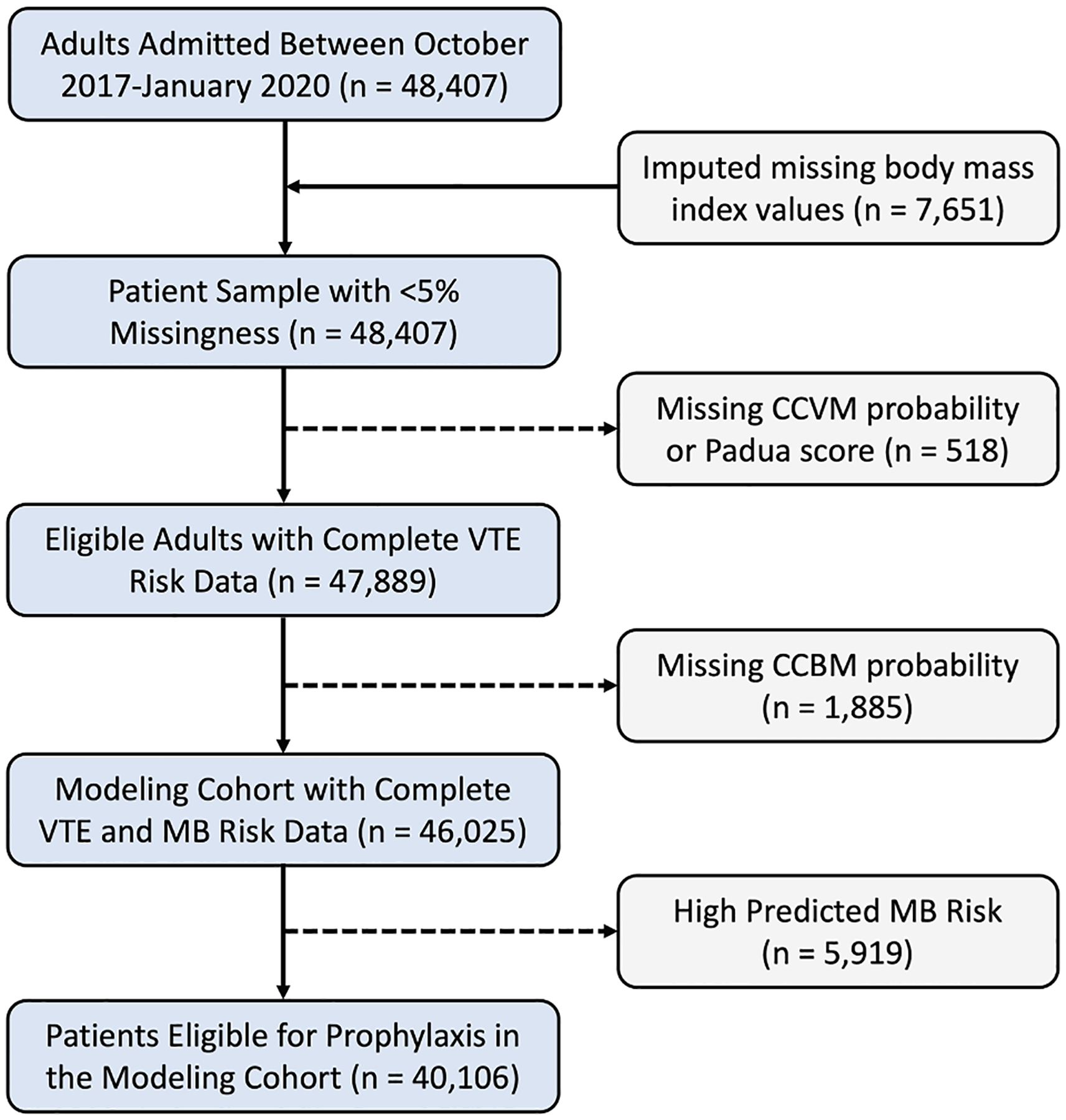
Study flow diagram showing multiple imputations and exclusions for key analytic variables. CCBM, Cleveland Clinic Bleeding Model; CCVM, Cleveland Clinic VTE Model; MB, major bleeding; VTE, venous thromboembolism.

A total of 224 of 434 physicians contacted completed the survey (response rate, 52%). After excluding 10 (4.5%) physicians who failed to answer Q1 or provided an ineligible response to Q2, our final sample contained 214 physicians. The patient cohort included 48 407 adult medical inpatients who met the eligibility criteria; 7651 (16%) had body mass index imputed using age, biological sex, race, heart failure status, and cancer status (see Supplementary Methods). After excluding 518 (1%) patients who were missing 1 or more variables, the analytic cohort comprised 47 889 patients (Figure 1). Within the analytic cohort, 29 388 (60.7%) patients received prophylaxis. There were 397 VTEs and 226 MBs among those who received prophylaxis, vs 104 VTEs and 71 MBs among those who did not receive prophylaxis.

### 3.2 | Comparator: decision analysis

**FIGURE 2:**
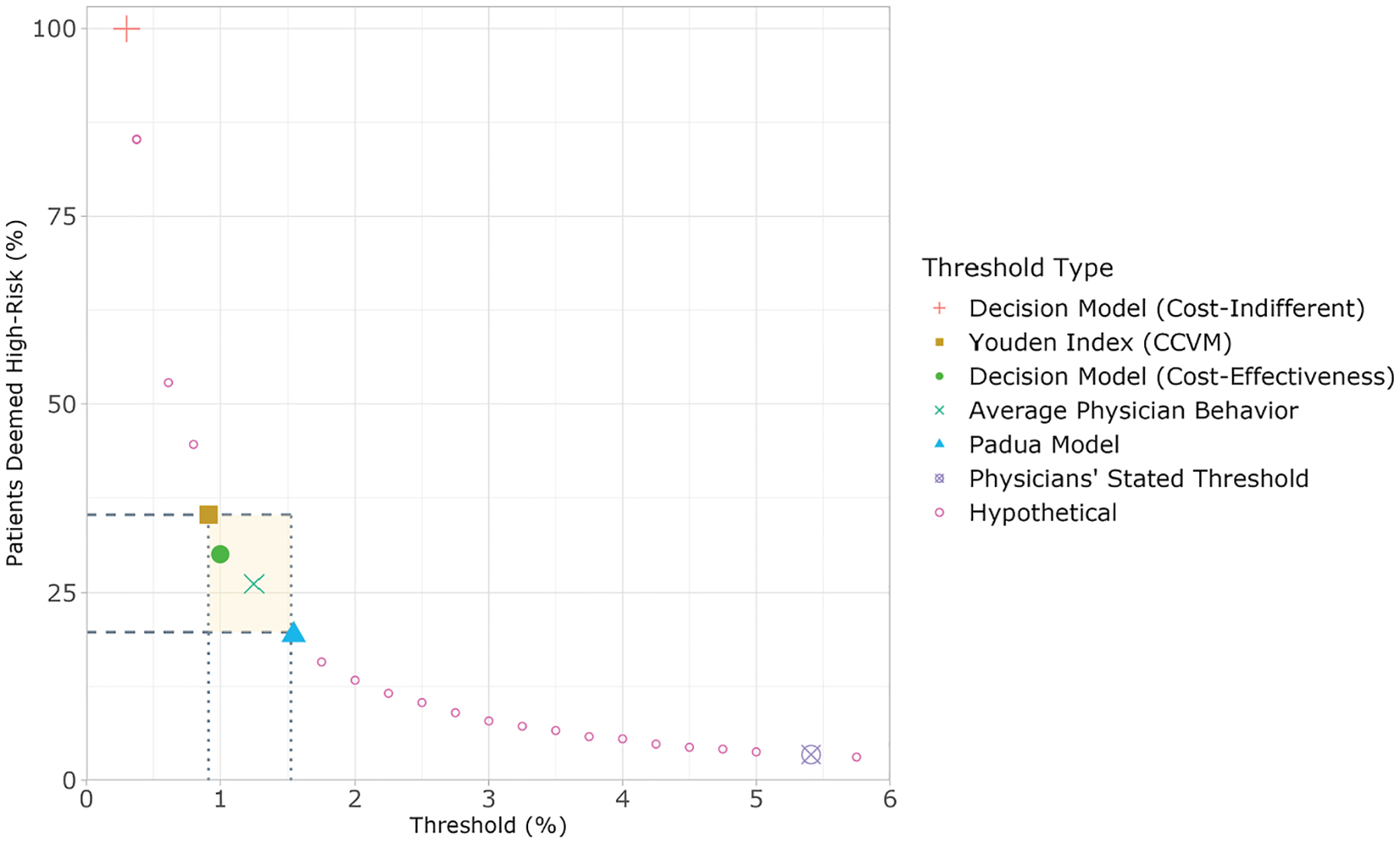
Empirical and theoretical venous thromboembolism risk thresholds vs the percentage of patients deemed high risk. The cost-effectiveness analysis produced a 1% threshold, with a corresponding high-risk percentage of 30.2%. Three other thresholds clustered between 0.9% and 1.5%, with corresponding high-risk percentages ranging from 19.3% to 35.2%, represented by the intersecting gray lines and shaded box. Two extreme thresholds would result in either 100% or <4% of patients being deemed high risk. Changing the threshold had the largest impact on the percentage of patients deemed high risk at the lower threshold range. CCVM, Cleveland Clinic VTE Model.

For CEA, prophylaxis had an incremental cost-effectiveness ratio of $100 000/QALY at a VTE probability of 1.0%. Ignoring costs, patients had a net benefit from prophylaxis if the probability of VTE was at least 0.3%. At the 1.0% threshold, 30% of patients would be considered high risk, whereas at the 0.3% threshold, 100% of patients would be considered high risk (Figure 2).

### 3.3 | Approach 1: maximize sensitivity and specificity of a logistic regression model (Youden Index)

The CCVM threshold, based on the Youden Index, was 0.9%. At this threshold, 35% of patients would be considered high risk.

### 3.4 | Approach 2: derive a probability from a point-based model (Padua)

A total of 4541 (9.5%) patients had a Padua score of exactly 4. Among these, 70 (1.5%) experienced VTE. At that threshold, 19% of patients would be considered high risk.

### 3.5 | Approach 3: survey physicians

The median physician considered a probability of 5% to be high risk; the mean across all responses was 5.3%, and the range was 1% to 10%. In response to Q2, the mean NNT was 86.6, the median was 50, and the range was 1 to 1000 after excluding the 2 outliers (see Supplementary Text and Supplementary Figures S1 and S2). We used the median response to Q2 to calculate physicians’ stated threshold, which was 5.4%, indicating that <4% of patients were high risk.

### 3.6 | Approach 4: examine physician behavior

Patients had an overall prophylaxis rate of 61%, which varied by predicted risk. The physicians’ point of equipoise, as measured by the logistic regression model (ie, the predicted risk at which 50% of patients would receive prophylaxis), was 1.25%, indicating that 26% of patients were high risk.

### 3.7 | Applying the risk thresholds

After excluding 1864 (3.9%) patients who were missing variables needed to calculate bleeding risk, the modeling cohort included 46 025 patients. Another 5919 (12.9%) patients were excluded because they were at high risk of MB and, therefore, ineligible for prophylaxis. Predicted VTE risks in the remaining cohort ranged from 0.31% to 45.3%, with a mean of 1.3% and a median of 0.7%.

**FIGURE 3:**
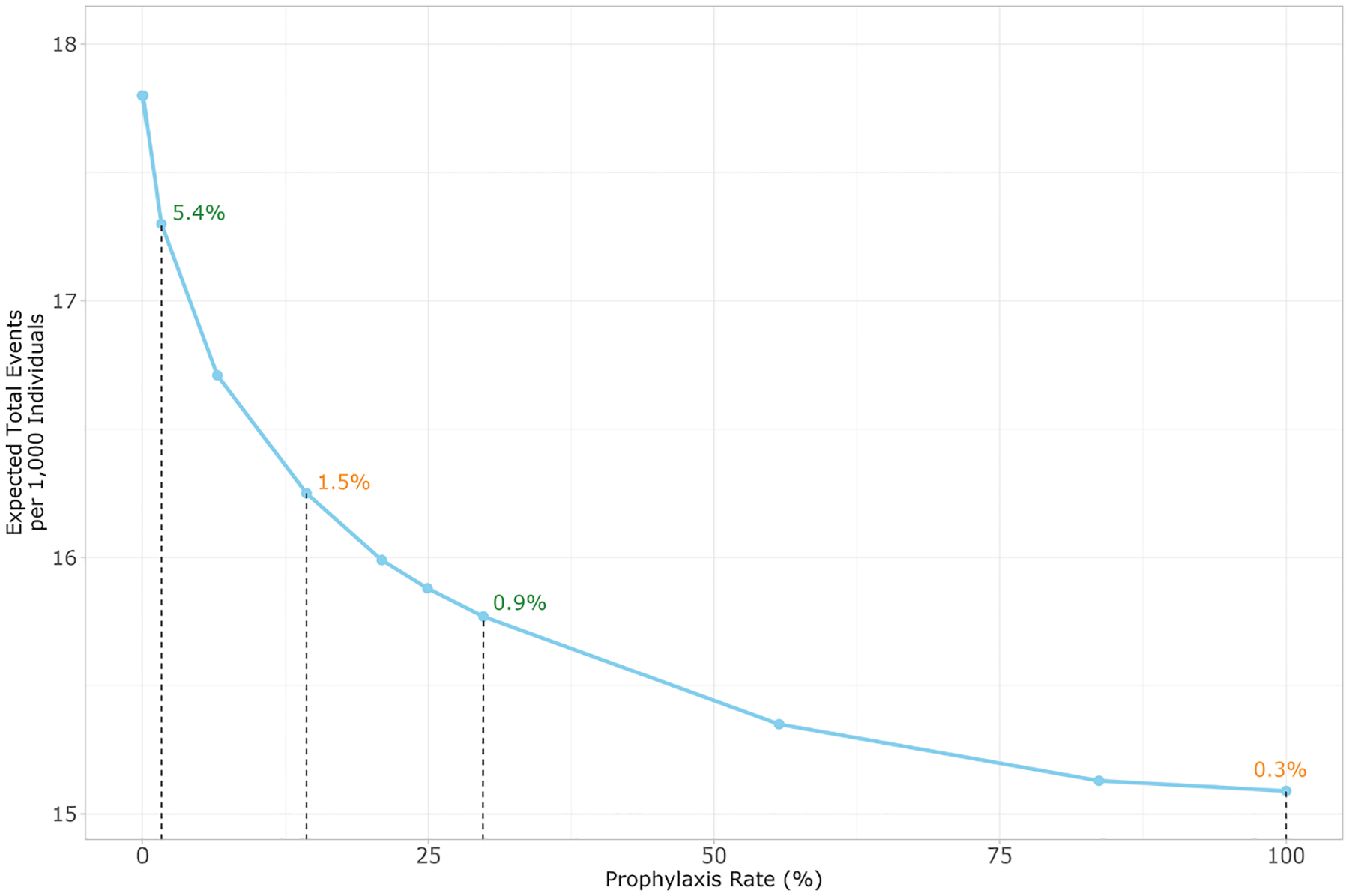
Threshold-guided prophylaxis rates among eligible patients (ie, excluding those at high risk of major bleeding) at each threshold vs expected total adverse event rates, combining venous thromboembolism and major bleeding (*N* = 46 025). Total adverse event rates ranged from 15.1 to 17.8 per 1000 individuals. Adverse events inversely decreased with threshold-guided prophylaxis rates. But with each successive increase in prophylaxis, the number needed to treat increased exponentially, indicating diminishing returns to adding more prophylaxis at each point. This indicates that venous thromboembolism prophylaxis is not equally efficient at all thresholds.

The Table lists, for each threshold, the expected rates of VTE, MB, and total adverse events per 1000 individuals. With no prophylaxis, we would expect 579 VTEs and 240 MBs in total. Applying thresholds ranging from 5.4% to 0.3%, the number of expected VTEs ranged from 392 to 555, and the expected MBs ranged from 303 to 242. More prophylaxis always reduced total adverse events, as the reduction in VTE more than offset the increase in MB. However, the incremental NNT increased exponentially (Figure 3). Moving from no prophylaxis to treating those with a risk of ≥5.4% required treating only 2% of eligible patients, with an NNT of 30. Lowering the threshold to 1.5% (a Padua score of 4) increased the percentage of patients receiving prophylaxis to 14%, with an NNT of 120. Further reducing it to 1% (based on the CEA) would require prophylaxis for 25% of patients (see Supplementary Table S4), with an NNT of 290. Compared with a threshold of 0.9% (the Youden index), providing prophylaxis to 100% of patients would require treating 1035 additional patients to prevent 1 additional adverse event.

## 4 | DISCUSSION

In this study, we compared decision analysis with 4 alternative approaches to derive an appropriate probability threshold for defining “high risk” for VTE and to guide prophylaxis in medical inpatients. We compared the resulting thresholds in terms of the percentage of patients who would be considered high risk and thus recommended prophylaxis, depending on bleeding risk. We found that thresholds varied by more than 10-fold across approaches, with corresponding percentages of patients identified as high risk varying by more than 30-fold. The inverse exponential relationship between threshold and the percentage of patients deemed high risk suggests that small changes in the threshold can have a substantial impact on this percentage, particularly in the most “active” decision-making area of the curve (indicated by the shaded box in Figure 2).

The upper limit of this range, a threshold of 1.5% based on the Padua score, would result in 14% of eligible patients being considered high risk after excluding those at high risk of bleeding. Although physicians, when asked directly, endorsed an even higher threshold (≥5.4%), it is unlikely that hospitals would accept such a low prophylaxis rate (<4%), especially after decades of quality improvement initiatives and hospital quality measures that favor prophylaxis [34–37]. We found multiple thresholds clustered within a tight range that offer a more practical target for efforts to curb prophylaxis overuse [20,38–40]. Physicians in our cohort displayed equipoise (treating 50% of patients) with a predicted probability of 1.25%. This was modestly higher than the optimal 1% threshold from the CEA decision model [30], which, in turn, was similar to the Youden Index (0.9%) of the CCVM [27].

In contrast to these practical thresholds, the more extreme thresholds we discovered are the least likely to be accepted in clinical practice. At the upper end of the threshold spectrum (≥5.4%), based on physicians’ stated thresholds, <4% of patients would receive prophylaxis, and the rate of adverse events would be high. At the lowest threshold (0.3%), based on the cost-indifferent decision model, all patients would be considered high risk. Although this would produce the fewest expected adverse events, universal prophylaxis is not recommended due to the inefficiency of providing prophylaxis to large numbers of patients who are unlikely to benefit [41], combined with the failure of this approach to meaningfully reduce VTE compared with targeted prophylaxis [42].

The more targeted approach represents a compromise between minimizing adverse events and wasting resources. Based on cost-effectiveness, the CEA model identified a 1% threshold. Lowering the threshold further simply increases the NNT; the efficiency of prophylaxis in reducing events decreased exponentially from the highest to the lowest threshold. Whereas increasing prophylaxis from 0% to 25% at the CEA threshold would require an incremental NNT of only 140, increasing it further from 25% to 100% resulted in an incremental NNT of 830. What is a reasonable NNT? The physicians we surveyed reported a median NNT of 50 (IQR, 20–100), indicating that 75% of physicians would reject a threshold below 1.5%. These findings highlight the inefficiency of universal prophylaxis and the need for a consistent approach based on VTE risk.

Standardization is important, but for most decisions, including VTE prophylaxis, the treatment threshold will vary depending on the clinical context [43]. Factors to consider include cost-effectiveness, resource availability, local risk factor distributions, and patient preferences. Even so, guideline committees should establish ranges of plausible thresholds that account for anticipated outcomes of risk stratification based on those thresholds [43]. Our findings suggest that for VTE, the probability threshold could reasonably range from 0.9% to 1.5%, resulting in 19% to 35% of patients being eligible for prophylaxis. This range is substantially lower than the average prophylaxis rates reported nationally by hospitals [44,45], with some reporting rates approaching 100% [42]. Evidence-based threshold selection could maximize the efficiency of clinical decision support by emphasizing the net benefit [46]. Our range of practical thresholds could inform the boundaries within which different organizations operate, based on their goals.

This study’s limitations include that the physician and patient samples were obtained from a single health system. But the percentage of patients determined to be high risk falls within ranges reported by others using a variety of tools [47]. We used a limited number of methods to determine potential thresholds; other methods could also be used [48]. Future work might empirically determine which approaches are best for meeting predefined hospital outcome targets or population health goals, such as VTE or MB incidence rates or recurrence rates, blood transfusion requirements for bleeding events, median length of hospital stay, readmission rates, or mortality rates. Additional work is also needed to quantify the different trade-offs between VTE and MB events in the context of concurrent risk assessment. In this study, we assumed that VTE and MB are equally harmful because studies show equivalent case-fatality rates within 3 months of anticoagulation [49]. Some physicians might argue for differential weighting based on clinical factors beyond mortality risk.

## 5 | CONCLUSION

We compared decision analysis with 4 alternative approaches to determine a probability threshold for identifying a high risk of VTE and evaluated risk stratification and clinical outcomes for each threshold. Thresholds varied widely, but those between 0.9% and 1.5% were most efficient in preventing VTE and MB events. Standardizing the definition of high risk could result in more efficient and appropriate patient-centered VTE prevention. Similar approaches could be applied to other common decisions for which risk assessment models are available.

## Supplementary Material

1

The online version contains supplementary material available at https://doi.org/10.1016/j.jtha.2026.02.004.

## Figures and Tables

**TABLE:** Expected rates of venous thromboembolism and major bleeding at each threshold, based on our model of threshold-guided prophylaxis.

VTE risk threshold approach	VTE risk threshold (%)	Patients deemed high risk (%)	Expected VTEs per 1000 individuals	Expected MBs per 1000 individuals	Expected total events per 1000 individuals	Incremental NNT
None	100	0	12.6	5.2	17.8	-
Physician survey	5.4	3.4	12.1	5.3	17.4	30
Padua model	1.5	19.3	10.8	5.5	16.3	120
Physician behavior	1.25	26.2	10.4	5.6	16.0	255
CEA	1.0	30.2	10.2	5.7	15.9	365
Youden Index	0.9	35.2	10.0	5.7	15.7	445
Cost-indifferent model	0.3	100	8.5	6.6	15.1	1,035

Patients with a high MB risk were considered ineligible for prophylaxis at any VTE threshold. Increasing prophylaxis would result in fewer VTEs but more MBs. Maximum prophylaxis (100% of eligible patients) would minimize the VTE rate, whereas 0% prophylaxis would minimize the MB rate. When summing VTE and MB rates, the minimum rate of 15.1 per 1000 individuals would be achieved at the cost-indifferent decision-analytic threshold (0.3%), with similar rates at thresholds ranging from 0.9% to 1.5%. However, the incremental NNT—the increase in the number of patients who would need prophylaxis to prevent 1 additional adverse event—was 2 to 35 times higher at the 0.3% threshold than at other thresholds.

CEA, cost-effectiveness analysis; MB, major bleeding; NNT, number needed to treat; VTE, venous thromboembolism.

## Data Availability

Deidentified summary data may be found in the supplementary material available with the online version of this article.

## References

[1] Beckman MG, Hooper WC, Critchley SE, Ortel TL. Venous thromboembolism: a public health concern. Am J Prev Med. 2010;38: S495–501. 10.1016/j.amepre.2009.12.017 20331949

[2] Anderson FA Jr, Zayaruzny M, Heit JA, Fidan D, Cohen AT. Estimated annual numbers of US acute-care hospital patients at risk for venous thromboembolism. Am J Hematol. 2007;82:777–82. 17626254 10.1002/ajh.20983

[3] Noboa S, Mottier D, Oger E, EPI-GETBO Study Group. Estimation of a potentially preventable fraction of venous thromboembolism: a community-based prospective study. J Thromb Haemost. 2006;4: 2720–2. 17100657 10.1111/j.1538-7836.2006.02196.x

[4] Heit JA, O’Fallon WM, Petterson TM, Lohse CM, Silverstein MD, Mohr DN, Melton LJ 3rd. Relative impact of risk factors for deep vein thrombosis and pulmonary embolism: a population-based study. Arch Intern Med. 2002;162:1245–8. 12038942 10.1001/archinte.162.11.1245

[5] Cohen AT, Davidson BL, Gallus AS, Lassen MR, Prins MH, Tomkowski W, Turpie AG, Egberts JF, Lensing AW, ARTEMIS Investigators. Efficacy and safety of fondaparinux for the prevention of venous thromboembolism in older acute medical patients: randomised placebo controlled trial. BMJ. 2006;332:325–9. 16439370 10.1136/bmj.38733.466748.7C PMC1363908

[6] Leizorovicz A, Cohen AT, Turpie AG, Olsson CG, Vaitkus PT, Goldhaber SZ, PREVENT Medical Thromboprophylaxis Study Group. Randomized, placebo-controlled trial of dalteparin for the prevention of venous thromboembolism in acutely ill medical patients. Circulation. 2004;110:874–9. 15289368 10.1161/01.CIR.0000138928.83266.24

[7] Samama MM, Cohen AT, Darmon JY, Desjardins L, Eldor A, Janbon C, Leizorovicz A, Nguyen H, Olsson CG, Turpie AG, Weisslinger N. A comparison of enoxaparin with placebo for the prevention of venous thromboembolism in acutely ill medical patients. Prophylaxis in Medical Patients with Enoxaparin Study Group. N Engl J Med. 1999;341:793–800. 10477777 10.1056/NEJM199909093411103

[8] Alhazzani W, Lim W, Jaeschke RZ, Murad MH, Cade J, Cook DJ. Heparin thromboprophylaxis in medical-surgical critically ill patients: a systematic review and meta-analysis of randomized trials. Crit Care Med. 2013;41:2088–98. 23782973 10.1097/CCM.0b013e31828cf104

[9] Datta I, Ball CG, Rudmik L, Hameed SM, Kortbeek JB. Complications related to deep venous thrombosis prophylaxis in trauma: a systematic review of the literature. J Trauma Manag Outcomes. 2010;4:1. 10.1186/1752-2897-4-1 20205800 PMC2823661

[10] Le Gal G, Agnelli G, Darius H, Kahn SR, Owaidah T, Rocha AT, Zhai Z, Khan I, Djoudi Y, Ponomareva E, Cohen AT. Event rates and risk factors for venous thromboembolism and major bleeding in a population of hospitalized adult patients with acute medical illness receiving enoxaparin thromboprophylaxis. Eur J Intern Med. 2024;121:48–55. 38030465 10.1016/j.ejim.2023.11.017

[11] Kearon C, Akl EA, Comerota AJ, Prandoni P, Bounameaux H, Goldhaber SZ, Nelson ME, Wells PS, Gould MK, Dentali F, Crowther M, Kahn SR. Antithrombotic therapy for VTE disease: antithrombotic therapy and prevention of thrombosis, 9th ed: American College of Chest Physicians evidence-based clinical practice guidelines. Chest. 2012;141:e419S–96S. 10.1378/chest.11-2301 22315268 PMC3278049

[12] Schünemann HJ, Cushman M, Burnett AE, Kahn SR, Beyer-Westendorf J, Spencer FA, Rezende SM, Zakai NA, Bauer KA, Dentali F, Lansing J, Balduzzi S, Darzi A, Morgano GP, Neumann I, Nieuwlaat R, Yepes-Nuñez JJ, Zhang Y, Wiercioch W. American Society of Hematology 2018 guidelines for management of venous thromboembolism: prophylaxis for hospitalized and nonhospitalized medical patients. Blood Adv. 2018;2:3198–225. 30482763 10.1182/bloodadvances.2018022954 PMC6258910

[13] Cushman M, Barnes GD, Creager MA, Diaz JA, Henke PK, Machlus KR, Nieman MT, Wolberg AS. American Heart Association Council on Peripheral Vascular Disease; Council on Arteriosclerosis, Thrombosis and Vascular Biology; Council on Cardiovascular and Stroke Nursing; Council on Clinical Cardiology; Council on Epidemiology and Prevention; and the International Society on Thrombosis and Haemostasis. Venous thromboembolism research priorities: a scientific statement from the American Heart Association and the International Society on Thrombosis and Haemostasis. Circulation. 2020;142:e85–94. 10.1161/CIR.0000000000000818 32776842

[14] Qaseem A, Chou R, Humphrey LL, Starkey M, Shekelle P, Clinical Guidelines Committee of the American College of Physicians. Venous thromboembolism prophylaxis in hospitalized patients: a clinical practice guideline from the American College of Physicians. Ann Intern Med. 2011;155:625–32. 22041951 10.7326/0003-4819-155-9-201111010-00011

[15] Woller SC, Stevens SM, Jones JP, Lloyd JF, Evans RS, Aston VT, Elliott CG. Derivation and validation of a simple model to identify venous thromboembolism risk in medical patients. Am J Med. 2011;124:947–54.e2. 10.1016/j.amjmed.2011.06.004 21962315

[16] Barbar S, Noventa F, Rossetto V, Ferrari A, Brandolin B, Perlati M, De Bon E, Tormene D, Pagnan A, Prandoni P. A risk assessment model for the identification of hospitalized medical patients at risk for venous thromboembolism: the Padua prediction score. J Thromb Haemost. 2010;8:2450–7. 20738765 10.1111/j.1538-7836.2010.04044.x

[17] Galbraith EM, Vautaw BM, Grzybowski M, Henke PK, Wakefield TW, Froehlich JB. Variation in physician deep vein thrombosis prophylaxis attitudes and practices at an academic tertiary care center. J Thromb Thrombolysis. 2010;30:419–25. 20174856 10.1007/s11239-010-0455-7

[18] Djulbegovic M, Chen K, Sureshanand S, Chaudhry S. Potential overuse of primary thromboprophylaxis in medical inpatients at low risk of venous thromboembolism. Blood. 2019;134:3385. 10.1182/blood-2019-131827 PMC8390711 33464465

[19] Grant PJ, Conlon A, Chopra V, Flanders SA. Use of venous thromboembolism prophylaxis in hospitalized patients. JAMA Intern Med. 2018;178:1122–4. 29800008 10.1001/jamainternmed.2018.2022 PMC6143102

[20] Pavon JM, Sloane RJ, Pieper CF, Colón-Emeric CS, Cohen HJ, Gallagher D, Morey MC, McCarty M, Ortel TL, Hastings SN. Poor adherence to risk stratification guidelines results in overuse of venous thromboembolism prophylaxis in hospitalized older adults. J Hosp Med. 2018;13:403–4. 29408946 10.12788/jhm.2916 PMC5984140

[21] Vickers AJ, Elkin EB. Decision curve analysis: a novel method for evaluating prediction models. Med Decis Making. 2006;26:565–74. 17099194 10.1177/0272989X06295361 PMC2577036

[22] Piovani D, Sokou R, Tsantes AG, Vitello AS, Bonovas S. Optimizing clinical decision making with decision curve analysis: insights for clinical investigators. Healthcare (Basel). 2023;11:2244. 10.3390/healthcare11162244 37628442 PMC10454914

[23] Jones C, Thornton J, Wyatt JC. Enhancing trust in clinical decision support systems: a framework for developers. BMJ Health Care Inform. 2021;28:e100247. 10.1136/bmjhci-2020-100247 PMC8183267 34088721

[24] Häfliger E, Kopp B, Darbellay Farhoumand P, Choffat D, Rossel JB, Reny JL, Aujesky D, Méan M, Baumgartner C. Risk assessment models for venous thromboembolism in medical inpatients. JAMA Netw Open. 2024;7:e249980. 10.1001/jamanetworkopen.2024.9980 38728035 PMC11087835

[25] Moumneh T, Riou J, Douillet D, Henni S, Mottier D, Tritschler T, Le Gal G, Roy PM. Validation of risk assessment models predicting venous thromboembolism in acutely ill medical inpatients: a cohort study. J Thromb Haemost. 2020;18:1398–407. 32168402 10.1111/jth.14796

[26] von Elm E, Altman DG, Egger M, Pocock SJ, Gøtzsche PC, Vandenbroucke JP, STROBE Initiative. The Strengthening the Reporting of Observational Studies in Epidemiology (STROBE) statement: guidelines for reporting observational studies. Lancet. 2007;370:1453–7. 18064739 10.1016/S0140-6736(07)61602-X

[27] Rothberg MB, Hamilton AC, Greene MT, Fox J, Lisheba O, Milinovich A, Gautier TN 4th, Kim P, Kaatz S, Hu B. Derivation and validation of a risk factor model to identify medical inpatients at risk for venous thromboembolism. Thromb Haemost. 2022;122: 1231–8. 34784645 10.1055/a-1698-6506

[28] Mittman BG, Sheehan M, Kojima L, Casacchia NJ, Lisheba O, Hu B, Pappas MA, Rothberg MB. Development and internal validation of the Cleveland Clinic Bleeding Model to predict major bleeding risk at admission in medical inpatients. J Thromb Haemost. 2024;22: 2855–63. 39002732 10.1016/j.jtha.2024.06.025

[29] Stekhoven DJ. missForest: nonparametric missing value imputation using Random Forest. R package version 1.6.1. 2025.

[30] Le P, Martinez KA, Pappas MA, Rothberg MB. A decision model to estimate a risk threshold for venous thromboembolism prophylaxis in hospitalized medical patients. J Thromb Haemost. 2017;15: 1132–41. 28371250 10.1111/jth.13687 PMC5712445

[31] Fluss R, Faraggi D, Reiser B. Estimation of the Youden Index and its associated cutoff point. Biom J. 2005;47:458–72. 16161804 10.1002/bimj.200410135

[32] Leizorovicz A, Mismetti P. Preventing venous thromboembolism in medical patients. Circulation. 2004;110:IV13–9. 10.1161/01.CIR.0000150640.98772.af. 15598642

[33] Alikhan R, Bedenis R, Cohen AT. Heparin for the prevention of venous thromboembolism in acutely ill medical patients (excluding stroke and myocardial infarction). Cochrane Database Syst Rev. 2014;2014: CD003747. 10.1002/14651858.CD003747.pub4 24804622 PMC6491079

[34] Amin A, Stemkowski S, Lin J, Yang G. Thromboprophylaxis rates in US medical centers: success or failure? J Thromb Haemost. 2007;5: 1610–6. 17663733 10.1111/j.1538-7836.2007.02650.x

[35] Cohen AT, Tapson VF, Bergmann JF, Goldhaber SZ, Kakkar AK, Deslandes B, Huang W, Zayaruzny M, Emery L, Anderson FA Jr, ENDORSE Investigators. Venous thromboembolism risk and prophylaxis in the acute hospital care setting (ENDORSE study): a multinational cross-sectional study. Lancet. 2008;371:387–94. 18242412 10.1016/S0140-6736(08)60202-0

[36] Keane MG, Ingenito EP, Goldhaber SZ. Utilization of venous thromboembolism prophylaxis in the medical intensive care unit. Chest. 1994;106:13–4. 8020258 10.1378/chest.106.1.13

[37] Hirsch DR, Ingenito EP, Goldhaber SZ. Prevalence of deep venous thrombosis among patients in medical intensive care. JAMA. 1995;274:335–7. 7609264

[38] Kocher B, Darbellay Farhoumand P, Pulver D, Kopp B, Choffat D, Tritschler T, Vollenweider P, Reny JL, Rodondi N, Aujesky D, Méan M, Baumgartner C. Overuse and underuse of thromboprophylaxis in medical inpatients. Res Pract Thromb Haemost. 2023;7: 102184. 10.1016/j.rpth.2023.102184 37745158 PMC10514554

[39] Djulbegovic M, Chen K, Sureshanand S, Chaudhry S. Overuse of primary thromboprophylaxis in medical inpatients at low risk of venous thromboembolism. J Gen Intern Med. 2021;36:2883–5. 33464465 10.1007/s11606-020-05993-x PMC8390711

[40] Deng J, Thomas L, Li H, Varughesekutty E, Shi Q, Sambharia M, Musto K, Petal G, Gordon E, Ruparella S, Bansal R. Overuse of DVT prophylaxis in medical inpatients. Blood. 2015;126:5563. 10.1182/blood.V126.23.5563.5563

[41] Djulbegovic B, Boylan A, Kolo S, Scheurer DB, Anuskiewicz S, Khaledi F, Youkhana K, Madgwick S, Maharjan N, Hozo I. Converting IMPROVE bleeding and VTE risk assessment models into a fast-and-frugal decision tree for optimal hospital VTE prophylaxis. Blood Adv. 2024;8:3214–24. 38621198 10.1182/bloodadvances.2024013166 PMC11225674

[42] Heit JA, Crusan DJ, Ashrani AA, Petterson TM, Bailey KR. Effect of a near-universal hospitalization-based prophylaxis regimen on annual number of venous thromboembolism events in the US. Blood. 2017;130:109–14. 28483763 10.1182/blood-2016-12-758995 PMC5510788

[43] Wynants L, van Smeden M, McLernon DJ, Timmerman D, Steyerberg EW, Van Calster B Topic Group ‘Evaluating diagnostic tests and prediction models’ of the STRATOS initiative. Three myths about risk thresholds for prediction models. BMC Med. 2019;17: 192. 10.1186/s12916-019-1425-3 31651317 PMC6814132

[44] Rothberg MB, Lahti M, Pekow PS, Lindenauer PK. Venous thromboembolism prophylaxis among medical patients at US hospitals. J Gen Intern Med. 2010;25:489–94. 20352366 10.1007/s11606-010-1296-y PMC2869415

[45] Flanders SA, Greene MT, Grant P, Kaatz S, Paje D, Lee B, Barron J, Chopra V, Share D, Bernstein SJ. Hospital performance for pharmacologic venous thromboembolism prophylaxis and rate of venous thromboembolism: a cohort study. JAMA Intern Med. 2014;174:1577–84. 25133488 10.1001/jamainternmed.2014.3384

[46] Meunier PY, Raynaud C, Guimaraes E, Gueyffier F, Letrilliart L. Barriers and facilitators to the use of clinical decision support systems in primary care: a mixed-methods systematic review. Ann Fam Med. 2023;21:57–69. 36690490 10.1370/afm.2908 PMC9870646

[47] Greene MT, Spyropoulos AC, Chopra V, Grant PJ, Kaatz S, Bernstein SJ, Flanders SA. Validation of risk assessment models of venous thromboembolism in hospitalized medical patients. Am J Med. 2016;129:1001.e9–18. 10.1016/j.amjmed.2016.03.031 27107925

[48] Unal I Defining an optimal cut-point value in ROC analysis: an alternative approach. Comput Math Methods Med. 2017;2017: 3762651. 10.1155/2017/3762651 28642804 PMC5470053

[49] Carrier M, Le Gal G, Wells PS, Rodger MA. Systematic review: case-fatality rates of recurrent venous thromboembolism and major bleeding events among patients treated for venous thromboembolism. Ann Intern Med. 2010;152:578–89. 20439576 10.7326/0003-4819-152-9-201005040-00008

