# Supplementary material for: What constitutes high risk for venous thromboembolism? Determining an appropriate threshold to initiate prophylaxis
Source: J Thromb Haemost. Author manuscript; Available in PMC 2026 Jul 9. (PMC13349235; doi:10.1016/j.jtha.2026.02.004)
Supplement: 1 [file NIHMS2192017-supplement-1.docx]

**SUPPLEMENTARY INFORMATION FOR: What Constitutes High Risk for Venous Thromboembolism? Determining an Appropriate Threshold to Initiate Prophylaxis**

Benjamin G Mittman, PhD^1,2,3^, Bo Hu, PhD^4^, Phuc Le, PhD^2^, Matthew A Pappas, MD, MPH^2,5^, Aaron Hamilton, MD^2,5^, Michael B Rothberg, MD, MPH^2^

^1^ Medical Scientist Training Program, School of Medicine, Case Western Reserve University, Cleveland, OH, USA

^2^ Center for Value-Based Care Research, Department of Internal Medicine and Geriatrics, Primary Care Institute, Cleveland Clinic, Cleveland, OH, USA

^3^ Department of Population and Quantitative Health Sciences, School of Medicine, Case Western Reserve University, Cleveland, OH, USA

^4^ Department of Biostatistics and Bioinformatics, Duke University, Durham, NC, USA

^5^ Department of Hospital Medicine, Integrated Hospital Care Institute, Cleveland Clinic, Cleveland, OH, USA

**Sections**

1. Supplementary Methods

2. Supplementary Text

3. Supplementary Figure S1-S2

4. Supplementary Table S1-S4

5. References

**Supplementary Methods**

***Missing Data and Multiple Imputation***

Missingness is inherent to EHR data. The reason for missingness is often unknown or random. In our dataset, several variables were missing for <5% of patients, with no know reason for missingness. For those variables (listed below), we removed patients missing at least one value to enable a complete case analysis, which generally does not introduce meaningful bias when the total level of missingness is low (i.e., below 5%). Only one variable, BMI, had missingness greater than 5%:

| **Variable** | **Percent of observations missing** |
| --- | --- |
| BMI | 15.8% |
| CCVM Probability | 1.07% |
| Mobility Score | 1.07% |
| Cancer  Heart Failure  Respiratory Failure  IBD  Recent Surgery  Ischemic Stroke  Thrombophilia  History of VTE  Infection  CVC or PICC Line  Mechanical Ventilation | 0.65%  0.65%  0.65%  0.65%  0.65%  0.65%  0.65%  0.65%  0.65%  0.65%  0.65% |
| All others | 0% |

To impute missing observations for BMI, we first performed univariate correlations between BMI and every other variable required to compute each patients’ Padua^1^ score. Age was the only other continuous variable assessed; all others were binary. Therefore, we considered the strongest correlations on the basis of the point estimate rather than using p-values, because p-value calculations are not robust for biserial correlations. Age, gender, race, heart failure, and cancer had the strongest univariate correlations with BMI (all |r| between 0.065 and 0.14), whereas the remaining variables had weaker correlations (all |r| < 0.045). Therefore, we performed multiple imputation using the R package missForest^2^ to fill in missing BMI values based on these five variables and removed patients missing any of the other analytic variables listed above.

***Cleveland Clinic Model Details***

The original Cleveland Clinic VTE Model^3^ (CCVM), published in 2022, was derived on a sample of 155,026 patients and validated on a temporal validation cohort of 53,210 patients. All patients were adult medical patients admitted to the Cleveland Clinic between 2011 and 2018. The derivation cohort had a 14-day VTE rate of 0.68%, and the temporal validation cohort had a VTE rate of 0.64%. However, in a sample of patients admitted more recently, between 2019 and 2020, the 14-day VTE rate was 1.1%. This sample underwent a more in-depth manual review process to identify outcomes, suggesting the 2011-2018 cohort was missing almost half of its true 14-day VTE outcomes.

To address this problem, we applied a large language model (LLM) that we recently trained and validated to automate the identification of VTE events from radiology reports. The full details of that work will be presented in a forthcoming publication, but the sensitivity of the LLM was approximately 98% and the positive predictive value was >85%. We applied the LLM to radiology reports available for all patients in the 2011-2018 cohort and identified potential VTEs that were missed in the original identification process outlined in the original publication.

In the original CCVM model development, the 2011-2018 cohort had a total of 1,426 VTEs, for a total 14-day VTE rate of 0.67%. From the 24.6% of the patients in the cohort who had at least one valid radiology report, 1,137 new VTEs were identified, which had been previously missed. Each of these VTEs was manually verified to be a new and acute VTE occurring within 14 days of admission. After adding these new VTEs to the previously identified outcomes, the 2011-2018 cohort had a new total of 2,563 VTEs for an overall 14-day VTE rate of 1.20%.

Using the updated set of outcomes for the same patient cohort, we refitted the multivariable logistic regression model reported in the original CCVM publication to obtain the new model intercept and coefficients. See Table A1 below for a full comparison between the ORs and 95% CIs of the original CCVM and refitted CCVM. There is good correspondence between the original and refitted effect sizes.

**Supplementary Text**

***Additional Results for Approach 3: Surveying Physicians***

*Q2 Outlier Assessment:* After excluding ineligible responses to Q2 such as non-numerical answers or answers less than one, we evaluated the full distribution of responses. NNT values ranged from one to one billion, with a median of 50 and a mean of greater than four million (Figure A1, Panel A). The one billion value was an overly influential outlier and not a clinically relevant number, so we then evaluated the distribution without that value. Responses ranged from 1 to 2,000, with a median of 50 and a mean of 95.5 (Figure A1, Panel B). However, only one participant responded 2,000, which was twice the next highest response of 1,000, which five physicians selected. Based on it being a lone overly influential value, we also considered the response of 2,000 to be an outlier and excluded it. Thus, the final set of responses ranged from 1 to 1,000, with a median of 50 and a mean of 86.6 (Figure A1, Panel C).


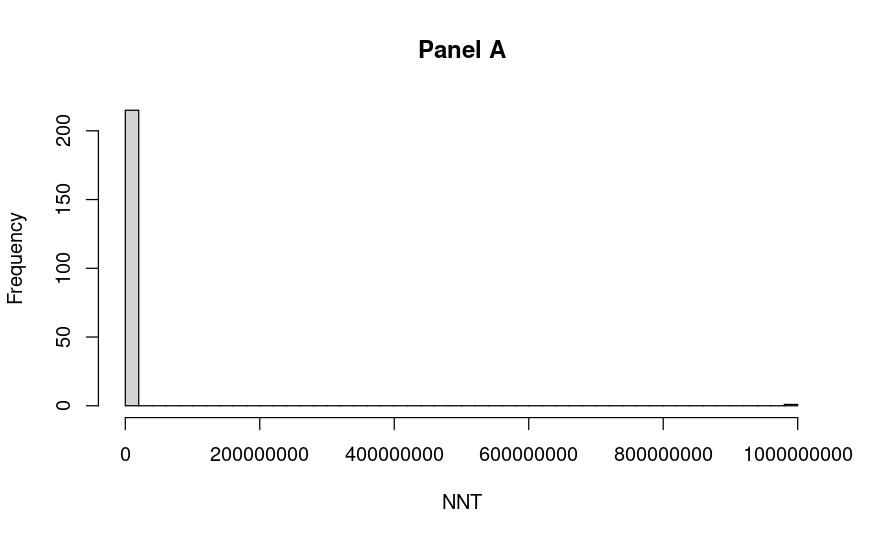

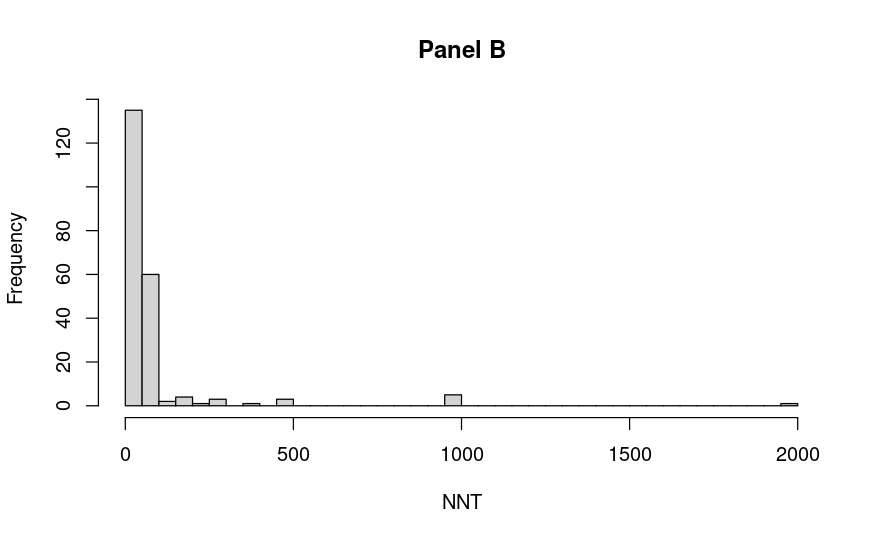

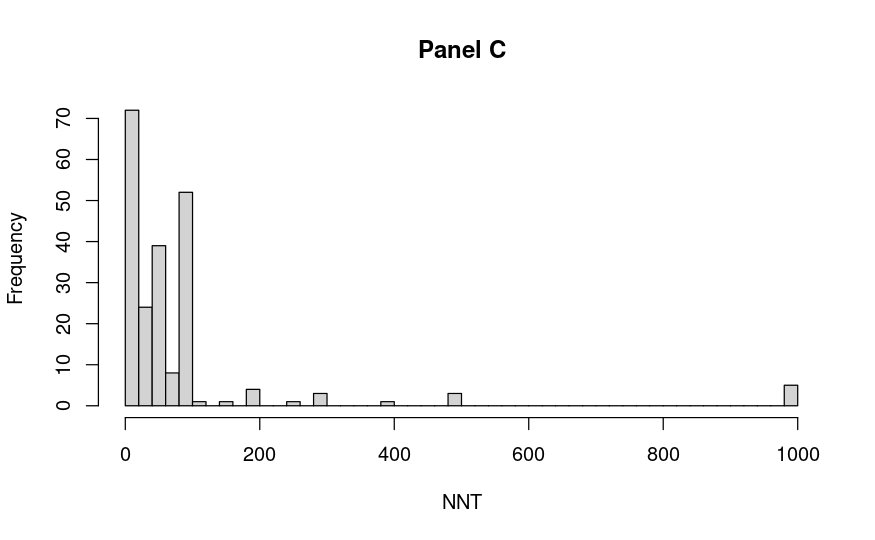


**Figure S1**. Histograms of Q2 responses including all eligible responses (Panel A), responses excluding the highest value of one billion (Panel B), and responses excluding both outliers of one billion and two thousand (Panel C).

*Correlation Between Q1 and Q2 Responses:* We also tested whether there was a significant correlation between physicians’ survey responses to Q1 and Q2, after excluding the two outliers from Q2. A Pearson’s correlation test showed no significant correlation between Q1 and Q2 responses (r = -0.045, p = 0.51; Figure A2).


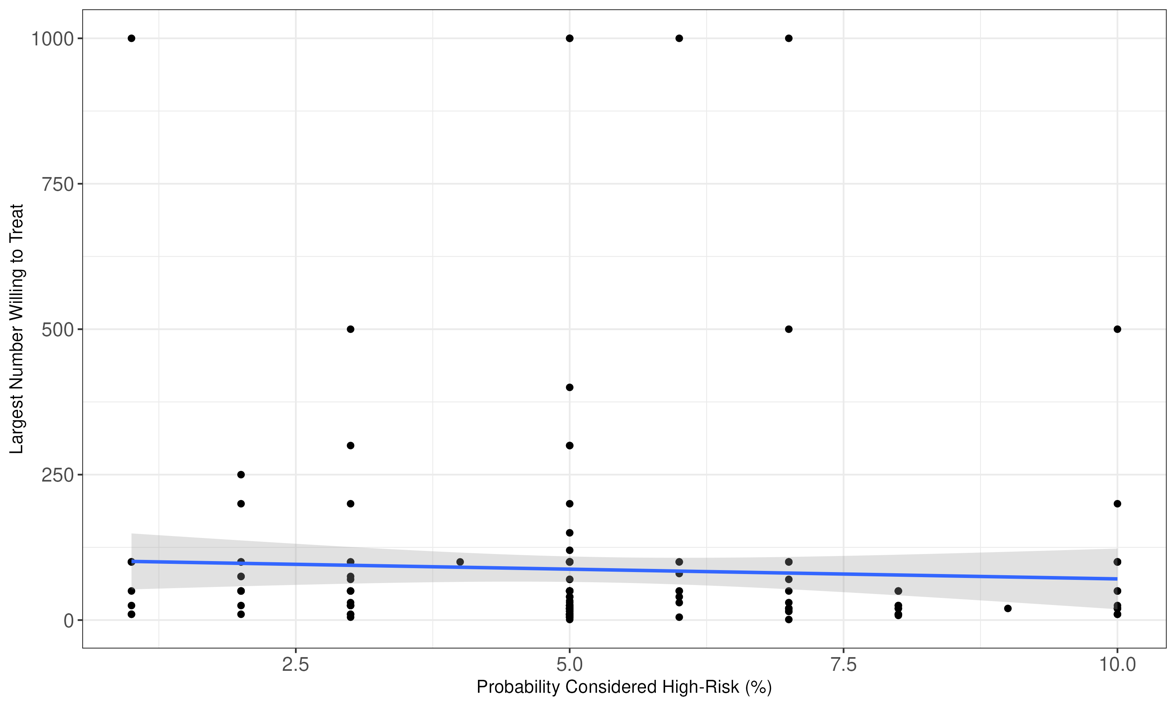


**Figure S2**. Surveyed physicians’ responses to Q1 (x-axis) versus Q2 (y-axis), excluding non-numerical answers (e.g., “cancer patients”) and two outliers (two thousand and one billion).

**Table S1**. STROBE Checklist.

|  | Item No | Recommendation | Page  No |
| --- | --- | --- | --- |
| **Title and abstract** | 1 | (*a*) Indicate the study’s design with a commonly used term in the title or the abstract | 2 |
|  |  | (*b*) Provide in the abstract an informative and balanced summary of what was done and what was found | 2 |
| Introduction | | | |
| Background/rationale | 2 | Explain the scientific background and rationale for the investigation being reported | 3-4 |
| Objectives | 3 | State specific objectives, including any prespecified hypotheses | 4 |
| Methods | | | |
| Study design | 4 | Present key elements of study design early in the paper | 4-5 |
| Setting | 5 | Describe the setting, locations, and relevant dates, including periods of recruitment, exposure, follow-up, and data collection | 4 |
| Participants | 6 | (*a*) *Cohort study*—Give the eligibility criteria, and the sources and methods of selection of participants. Describe methods of follow-up  *Case-control study*—Give the eligibility criteria, and the sources and methods of case ascertainment and control selection. Give the rationale for the choice of cases and controls  *Cross-sectional study*—Give the eligibility criteria, and the sources and methods of selection of participants | 4-5 |
|  |  | (*b*) *Cohort study*—For matched studies, give matching criteria and number of exposed and unexposed  *Case-control study*—For matched studies, give matching criteria and the number of controls per case | 4-5 |
| Variables | 7 | Clearly define all outcomes, exposures, predictors, potential confounders, and effect modifiers. Give diagnostic criteria, if applicable | 5 |
| Data sources/ measurement | 8* | For each variable of interest, give sources of data and details of methods of assessment (measurement). Describe comparability of assessment methods if there is more than one group | 5-7 |
| Bias | 9 | Describe any efforts to address potential sources of bias | 5-8 |
| Study size | 10 | Explain how the study size was arrived at | 5,9 |
| Quantitative variables | 11 | Explain how quantitative variables were handled in the analyses. If applicable, describe which groupings were chosen and why | 5 |
| Statistical methods | 12 | (*a*) Describe all statistical methods, including those used to control for confounding | 5-9 |
|  |  | (*b*) Describe any methods used to examine subgroups and interactions | 6-9 |
|  |  | (*c*) Explain how missing data were addressed | 5 |
|  |  | (*d*) *Cohort study*—If applicable, explain how loss to follow-up was addressed  *Case-control study*—If applicable, explain how matching of cases and controls was addressed  *Cross-sectional study*—If applicable, describe analytical methods taking account of sampling strategy | N/A |
|  |  | (*e*) Describe any sensitivity analyses | N/A |

Continued on next page

| Results | | | |
| --- | --- | --- | --- |
| Participants | 13* | (a) Report numbers of individuals at each stage of study—eg numbers potentially eligible, examined for eligibility, confirmed eligible, included in the study, completing follow-up, and analysed | 9 |
|  |  | (b) Give reasons for non-participation at each stage | 9 |
|  |  | (c) Consider use of a flow diagram | 21 |
| Descriptive data | 14* | (a) Give characteristics of study participants (eg demographic, clinical, social) and information on exposures and potential confounders | 9 |
|  |  | (b) Indicate number of participants with missing data for each variable of interest | 9; Supp. |
|  |  | (c) *Cohort study*—Summarise follow-up time (eg, average and total amount) | N/A |
| Outcome data | 15* | *Cohort study*—Report numbers of outcome events or summary measures over time | 9 |
|  |  | *Case-control study—*Report numbers in each exposure category, or summary measures of exposure | N/A |
|  |  | *Cross-sectional study—*Report numbers of outcome events or summary measures | 9 |
| Main results | 16 | (*a*) Give unadjusted estimates and, if applicable, confounder-adjusted estimates and their precision (eg, 95% confidence interval). Make clear which confounders were adjusted for and why they were included | 9-11 |
|  |  | (*b*) Report category boundaries when continuous variables were categorized | 10 |
|  |  | (*c*) If relevant, consider translating estimates of relative risk into absolute risk for a meaningful time period | N/A |
| Other analyses | 17 | Report other analyses done—eg analyses of subgroups and interactions, and sensitivity analyses | 10-11 |
| Discussion | | | |
| Key results | 18 | Summarise key results with reference to study objectives | 11-13 |
| Limitations | 19 | Discuss limitations of the study, taking into account sources of potential bias or imprecision. Discuss both direction and magnitude of any potential bias | 13 |
| Interpretation | 20 | Give a cautious overall interpretation of results considering objectives, limitations, multiplicity of analyses, results from similar studies, and other relevant evidence | 12-13 |
| Generalisability | 21 | Discuss the generalisability (external validity) of the study results | 12-13 |
| Other information | | | |
| Funding | 22 | Give the source of funding and the role of the funders for the present study and, if applicable, for the original study on which the present article is based | 14 |

*Give information separately for cases and controls in case-control studies and, if applicable, for exposed and unexposed groups in cohort and cross-sectional studies.

**Note:** An Explanation and Elaboration article discusses each checklist item and gives methodological background and published examples of transparent reporting. The STROBE checklist is best used in conjunction with this article (freely available on the Web sites of PLoS Medicine at http://www.plosmedicine.org/, Annals of Internal Medicine at http://www.annals.org/, and Epidemiology at http://www.epidem.com/). Information on the STROBE Initiative is available at www.strobe-statement.org.

**Table S2**. Cleveland Clinic Model odds ratios, comparing the original^*^ versus the refitted^†^ model.

| Variable | Original OR | Original OR  (95% CI) | | Refitted OR | Refitted OR  (95% CI) | |
| --- | --- | --- | --- | --- | --- | --- |
| Ambulation status | | | | | | |
| Walks frequently | Reference | - | - | Reference | - | - |
| Walks occasionally | 1.42 | 1.18 | 1.70 | 1.53 | 1.34 | 1.76 |
| Chairfast | 2.10 | 1.67 | 2.65 | 2.49 | 2.10 | 2.96 |
| Bedbound | 2.07 | 1.67 | 2.55 | 2.76 | 2.36 | 3.23 |
| Active cancer | 1.72 | 1.50 | 1.98 | 1.93 | 1.75 | 2.13 |
| Mechanical ventilation | 1.28 | 1.04 | 1.58 | 1.50 | 1.29 | 1.75 |
| Respiratory failure | 1.64 | 1.36 | 1.97 | 1.54 | 1.35 | 1.75 |
| Inflammatory bowel disease | 1.79 | 1.31 | 2.45 | 1.54 | 1.20 | 1.96 |
| Decubitus ulcer | 1.62 | 1.28 | 2.06 | 1.43 | 1.21 | 1.68 |
| Recent surgery | 1.61 | 1.26 | 2.05 | 1.66 | 1.39 | 1.98 |
| Ischemic stroke | 1.39 | 1.09 | 1.76 | 1.67 | 1.43 | 1.95 |
| Thrombophilia | 2.13 | 1.21 | 3.74 | 1.73 | 1.06 | 2.66 |
| Central venous catheter^‡^ | 3.03 | 2.61 | 3.52 | 2.20 | 1.94 | 2.50 |
| History of VTE | 2.28 | 1.86 | 2.81 | 2.23 | 1.94 | 2.54 |
| Acute infection | 1.78 | 1.55 | 2.06 | 1.82 | 1.66 | 2.01 |

Abbreviations: OR, odds ratio; CI, confidence interval; VTE, venous thromboembolism.

*Original model intercept: –6.216

†Refitted model intercept: –4.746

‡Includes peripherally inserted central catheter.

**Table S3**. Complete set of input values and results from calculations of expected VTE and MB outcomes, listed for each VTE threshold considered.

| Model Term | 100% | 5.4% | 1.5% | 1.25% | 1.0% | 0.9% | 0.3% |
| --- | --- | --- | --- | --- | --- | --- | --- |
| ${AV}_{[HV,LB]}$ | NaN | 0.0832 | 0.0315 | 0.0259 | 0.0235 | 0.0212 | 0.0101 |
| $N_{[HV,LB]}$ | 0 | 627 | 5715 | 8384 | 9987 | 11944 | 40106 |
| ${RR}_{VTE}$ | 0.54 | 0.54 | 0.54 | 0.54 | 0.54 | 0.54 | 0.54 |
| ${AV}_{[LV,LB]}$ | 0.0101 | 0.00895 | 0.00655 | 0.00593 | 0.00565 | 0.00541 | NaN |
| $N_{[LV,LB]}$ | 40106 | 39479 | 34391 | 31722 | 30119 | 28162 | 0 |
| ${AV}_{[HB]}$ | 0.0293 | 0.0293 | 0.0293 | 0.0293 | 0.0293 | 0.0293 | 0.0293 |
| $N_{[HB]}$ | 5919 | 5919 | 5919 | 5919 | 5919 | 5919 | 5919 |
| VTE Total | 578.78 | 554.77 | 495.86 | 478.88 | 470.62 | 462.32 | 392.30 |
| VTE Rate* | 12.58 | 12.05 | 10.77 | 10.40 | 10.23 | 10.04 | 8.52 |
| ${AB}_{[HV,LB]}$ | NaN | 0.00382 | 0.00322 | 0.00313 | 0.00304 | 0.00304 | 0.00239 |
| $N_{[HV,LB]}$ | 0 | 627 | 5715 | 8384 | 9987 | 11944 | 40106 |
| ${RR}_{MB}$ | 1.65 | 1.65 | 1.65 | 1.65 | 1.65 | 1.65 | 1.65 |
| ${AB}_{[LV,LB]}$ | 0.00239 | 0.00237 | 0.00225 | 0.00219 | 0.00217 | 0.00211 | NaN |
| $N_{[LV,LB]}$ | 40106 | 39479 | 34391 | 31722 | 30119 | 28162 | 0 |
| ${AB}_{[HB]}$ | 0.0244 | 0.0244 | 0.0244 | 0.0244 | 0.0244 | 0.0244 | 0.0244 |
| $N_{[HB]}$ | 5919 | 5919 | 5919 | 5919 | 5919 | 5919 | 5919 |
| MB Total | 240.19 | 241.75 | 252.17 | 257.27 | 259.95 | 263.83 | 302.48 |
| MB Rate* | 5.22 | 5.25 | 5.48 | 5.59 | 5.65 | 5.73 | 6.57 |
| VTE + MB Total | 17.80 | 17.30 | 16.25 | 15.99 | 15.88 | 15.77 | 15.09 |

Note: all model terms are described in the main manuscript beneath the full equations, in the Methods.

*Rate is expressed as the number of events per 1,000 individuals, calculated according to the formula:

$$Rate=\frac{Event Total}{46,025}\times1,000$$

**Table S4**. High-risk percentages and ideal prophylaxis rates for each threshold.

| VTE Risk Threshold (%) | Patients Deemed High-Risk in Full Cohort (%)^*^ | Ideal Prophylaxis Rate Among All Patients (%)^†^ | Ideal Prophylaxis Rate Among Eligible Patients (%)^‡^ |
| --- | --- | --- | --- |
| 100 | 0 | 0 | 0 |
| 5.4 | 3.4 | 1.4 | 1.6 |
| 1.5 | 19.3 | 12.4 | 14.3 |
| 1.25 | 26.2 | 18.2 | 20.9 |
| 1.0 | 30.2 | 21.7 | 24.9 |
| 0.9 | 35.2 | 26.0 | 29.8 |
| 0.3 | 100 | 87.1 | 100 |

Note: VTE, venous thromboembolism. MB, major bleeding.

*The percentage of patients in the full cohort (N = 47,889) considered high-risk for VTE by the CCVM

†The percentage of patients in the modeling cohort (N = 46,025) who would ideally receive prophylaxis

‡The percentage of patients in the modeling cohort considered eligible for prophylaxis based on MB risk (N = 40,106) who would ideally receive prophylaxis

**References**

1. Barbar S, Noventa F, Rossetto V, et al. A risk assessment model for the identification of hospitalized medical patients at risk for venous thromboembolism: The Padua Prediction Score. *Journal of Thrombosis and Haemostasis*. 2010;8(11):2450-2457. doi:10.1111/j.1538-7836.2010.04044.x

2. Stekhoven DJ, Bühlmann P. MissForest—non-parametric missing value imputation for mixed-type data. *Bioinformatics*. 2012;28(1):112-118. doi:10.1093/BIOINFORMATICS/BTR597

3. Rothberg MB, Hamilton AC, Greene MT, et al. Derivation and Validation of a Risk Factor Model to Identify Medical Inpatients at Risk for Venous Thromboembolism. *Thromb Haemost*. 2022;122(7):1231-1238. doi:10.1055/A-1698-6506/ID/JR210284-2/BIB
